# Spectrum, risk factors and outcomes of neurological and psychiatric complications of COVID-19: a UK-wide cross-sectional surveillance study

**DOI:** 10.1093/braincomms/fcab168

**Published:** 2021-07-22

**Authors:** Amy L Ross Russell, Marc Hardwick, Athavan Jeyanantham, Laura M White, Saumitro Deb, Girvan Burnside, Harriet M Joy, Craig J Smith, Thomas A Pollak, Timothy R Nicholson, Nicholas W S Davies, Hadi Manji, Ava Easton, Stephen Ray, Michael S Zandi, Jonathan P Coles, David K Menon, Aravinthan Varatharaj, Beth McCausland, Mark A Ellul, Naomi Thomas, Gerome Breen, Stephen Keddie, Michael P Lunn, John P S Burn, Graziella Quattrocchi, Luke Dixon, Claire M Rice, George Pengas, Rustam Al-Shahi Salman, Alan Carson, Eileen M Joyce, Martin R Turner, Laura A Benjamin, Tom Solomon, Rachel Kneen, Sarah Pett, Rhys H Thomas, Benedict D Michael, Ian Galea

**Affiliations:** 1 NIHR Southampton Clinical Research Facility and Biomedical Research Centre, University Hospital Southampton NHS Foundation Trust, Southampton SO16 6YD, UK; 2 Department of Neurology, Wessex Neurological Centre, University Hospital Southampton NHS Foundation Trust, Southampton SO16 6YD, UK; 3 Clinical Neurosciences, Clinical and Experimental Sciences, Faculty of Medicine, University of Southampton, Southampton SO16 6YD, UK; 4 Liverpool University Hospitals NHS Foundation Trust, Liverpool, L9 7AL, UK; 5 Liverpool Clinical Trials Centre, University of Liverpool, Liverpool, L3 5TR, UK; 6 Department of Health Data Science, University of Liverpool, Liverpool, L69 3BX, UK; 7 Neuroradiology Department, Wessex Neurological Centre, University Hospital Southampton NHS Foundation Trust, Southampton, SO16 6YD, UK; 8 Manchester Centre for Clinical Neurosciences, Geoffrey Jefferson Brain Research Centre, Manchester Academic Health Science Centre, Salford Royal Foundation Trust, Salford, M6 8HD, UK; 9 Division of Cardiovascular Sciences, Lydia Becker Institute for Immunology and Inflammation, University of Manchester, Manchester, M13 9PL, UK; 10 Institute of Psychiatry, Psychology, and Neuroscience, King’s College London, London, SE5 8AF, UK; 11 Chelsea and Westminster NHS Foundation Trust, London, London, SW10 9NH, UK; 12 MRC Centre for Neuromuscular Diseases, National Hospital for Neurology, London, WC1N 3BG, UK; 13 UCL Queen Square Institute of Neurology, University College London, London, WC1N 3BG, UK; 14 Encephalitis Society, Malton, Malton, YO17 7DT, UK; 15 Department of Clinical Infection Microbiology and Immunology, Institute of Infection, Veterinary, and Ecological Sciences, University of Liverpool, Liverpool, L7 3EA, UK; 16 The National Institute for Health Research Health Protection Research Unit for Emerging and Zoonotic Infections, University of Liverpool, Liverpool, L69 7BE, UK; 17 Division of Anaesthesia, Department of Medicine, University of Cambridge, Cambridge, CB2 0SP, UK; 18 Memory Assessment and Research Centre, Moorgreen Hospital, Southern Health Foundation Trust, Southampton, SO40 2RZ, UK; 19 Department of Neurology, The Walton Centre NHS Foundation Trust, Liverpool, L9 7LJ, UK; 20 Translational and Clinical Research Institute, Newcastle University, Newcastle, NE1 7RU, UK; 21 Wellcome Centre for Mitochondrial Research, Newcastle University, Newcastle, NE2 4HH, UK; 22 Department of Social Genetic and Developmental Psychiatry, King’s College London, London, SE5 8AF, UK; 23 Department of Neuromuscular Diseases, University College London, London, WC1N 3BG, UK; 24 National Hospital for Neurology and Neurosurgery, University College London Hospitals NHS Foundation Trust, London, WC1N 3BG, UK; 25 Rehabilitation Department, Poole Hospital, University Hospitals Dorset NHS Foundation Trust, Poole, BH15 2JB, UK; 26 Department of Neurology, North Middlesex University Hospital NHS Trust, London, N18 1QX, UK; 27 Department of Neuroradiology, Imperial College NHS Healthcare Trust, London, W2 1NY, UK; 28 Department of Neurology, Southmead Hospital, North Bristol NHS Trust, Bristol, S10 5NB, UK; 29 Translational Health Sciences, Bristol Medical School, University of Bristol, Bristol, BS8 1TH, UK; 30 Centre for Clinical Brain Sciences, University of Edinburgh, Edinburgh, EH16 4SB, UK; 31 Nuffield Department of Clinical Neurosciences, University of Oxford, Oxford, OX3 9DU, UK; 32 Laboratory of Molecular and Cell Biology, UCL, Gower St, King’s Cross, London, London, WC1E 6BT, UK; 33 Department of Neurology, Alder Hey Children’s NHS Foundation Trust, Liverpool, Liverpool, L14 5AB, UK; 34 Medical Research Council Clinical Trials Unit, Institute of Clinical Trials and Methodology, University College London, London, WC1V 6LJ, UK; 35 Institute for Global Health, University College London, London, WC1N 1EH, UK; 36 Department of Neurology, Royal Victoria Infirmary, Newcastle, NE1 4LP, UK

**Keywords:** COVID-19, SARS-CoV-2, encephalopathy, stroke, neurology

## Abstract

SARS-CoV-2 is associated with new-onset neurological and psychiatric conditions. Detailed clinical data, including factors associated with recovery, are lacking, hampering prediction modelling and targeted therapeutic interventions. In a UK-wide cross-sectional surveillance study of adult hospitalized patients during the first COVID-19 wave, with multi-professional input from general and sub-specialty neurologists, psychiatrists, stroke physicians, and intensivists, we captured detailed data on demographics, risk factors, pre-COVID-19 Rockwood frailty score, comorbidities, neurological presentation and outcome. *A priori* clinical case definitions were used, with cross-specialty independent adjudication for discrepant cases. Multivariable logistic regression was performed using demographic and clinical variables, to determine the factors associated with outcome. A total of 267 cases were included. Cerebrovascular events were most frequently reported (131, 49%), followed by other central disorders (95, 36%) including delirium (28, 11%), central inflammatory (25, 9%), psychiatric (25, 9%), and other encephalopathies (17, 7%), including a severe encephalopathy (*n* = 13) not meeting delirium criteria; and peripheral nerve disorders (41, 15%). Those with the severe encephalopathy, in comparison to delirium, were younger, had higher rates of admission to intensive care and a longer duration of ventilation. Compared to normative data during the equivalent time period prior to the pandemic, cases of stroke in association with COVID-19 were younger and had a greater number of conventional, modifiable cerebrovascular risk factors. Twenty-seven per cent of strokes occurred in patients <60 years. Relative to those >60 years old, the younger stroke patients presented with delayed onset from respiratory symptoms, higher rates of multi-vessel occlusion (31%) and systemic thrombotic events. Clinical outcomes varied between disease groups, with cerebrovascular disease conferring the worst prognosis, but this effect was less marked than the pre-morbid factors of older age and a higher pre-COVID-19 frailty score, and a high admission white cell count, which were independently associated with a poor outcome. In summary, this study describes the spectrum of neurological and psychiatric conditions associated with COVID-19. In addition, we identify a severe COVID-19 encephalopathy atypical for delirium, and a phenotype of COVID-19 associated stroke in younger adults with a tendency for multiple infarcts and systemic thromboses. These clinical data will be useful to inform mechanistic studies and stratification of patients in clinical trials.

## Teaser

Ross Russell et al. report novel details of COVID-19 neurological complications: multiple overlapping diagnoses in the same patients, a severe encephalopathy atypical for delirium, and a lowered threshold for stroke in younger adults. Higher age, pre-morbid frailty score and white cell count predicted clinical outcome.

## Graphical Abstract

**Graphical Abstract:**
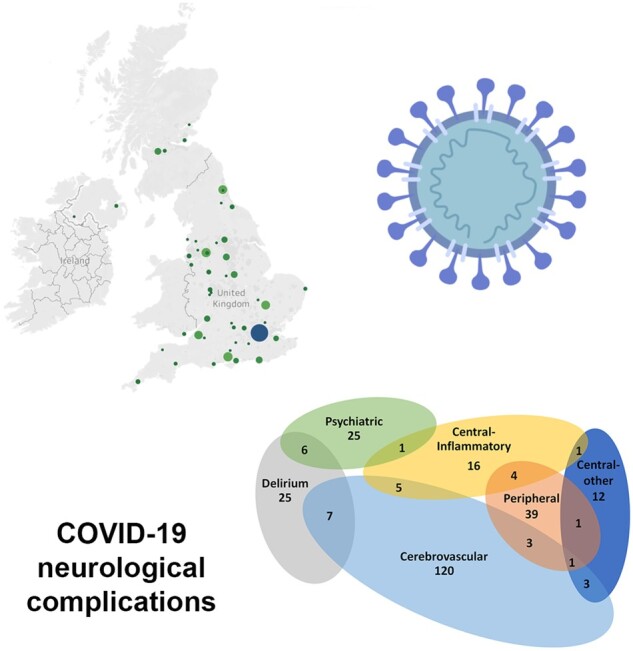

## Introduction

COVID-19 causes a multi-system disorder associated with a broad spectrum of neurological and neuropsychiatric complications.^1^^,^^2^ Mild disease has been associated with neurological *symptoms*, such as headache, anosmia and ageusia^1^^,^^3^ without major neurological complications.^4^ Approximately 10–25% of patients hospitalized with COVID-19 present with or develop a significant neurological *disorder*,^4–8^ the risk of which may increase with disease severity.^1^^,^^9^ Complications may reflect para- or post-infectious central and peripheral immune-mediated syndromes, or rarely direct CNS infection.^10^^,^^11^ We are at the early stages of understanding the impact of these neurological complications of COVID-19.

As neurological complications are varied and occur throughout the disease course, multiple mechanisms have been proposed. These may include direct viral infection of endothelium via angiotensin converting enzyme-2 receptors, systemic inflammation resulting in coagulopathy, cytokine toxicity, blood–brain barrier disruption, antibody and cell-mediated autoimmunity and consequences of prolonged severe illness.^2^^,^^12–15^ These suggested pathological processes may co-exist, act synergistically and occur simultaneously in different parts of the nervous system, causing overlapping clinical presentations.

Studies reporting neurological complications of COVID-19 have successfully met the pressing need to disseminate data rapidly to inform pandemic management and research efforts. However, this speed has limited geographical reach, so there is a paucity of nationwide studies and limited detailed clinical diagnostic and prognostic information. This is further hampered by a lack of unified diagnostic criteria and under-appreciation of overlapping presentations. Consequently, the factors predicting recovery remain poorly understood.

To address these gaps, we conducted a UK-wide surveillance study of neurological and psychiatric complications of COVID-19 (March–October 2020). National and cross-specialty recruitment was conducted to identify common and rarer presentations, and incorporated rigorous clinical case definitions to evaluate overlapping neurological presentations and determine the factors associated with recovery. In this paper, we first deliver an overview of the main neurological and psychiatric manifestations encountered. Then we present more detail on each category of disorder and perform analyses to try to deliver insight into prognosis and underlying disease mechanisms.

## Materials and methods

### Study design

Physicians were invited to complete standardized electronic Case Record Forms (CRFs) by the five major professional neuroscience associations in the UK (Association of British Neurologists, British Association of Stroke Physicians, Royal College of Psychiatrists, the Neuro Anaesthesia and Critical Care Society, and the Intensive Care Society). This study was approved by the University of Liverpool (UoL #7725/2020) and the University of Southampton (ERGO #56504). The British Peripheral Nerve Society’s surveillance study for Guillain–Barré syndrome was performed independently,^16^ but the case definitions and data fields were aligned to enable inclusion. Four cases were published as single case studies (Supplementary Table 1). The UK Health Research Authority advised that the study did not require review by a NHS Research Ethics Committee as this was a surveillance study with non-identifiable information.

The CRF included demographics, evidence of SARS-CoV-2 infection, neurological and non-neurological clinical features, pre-morbid Rockwood frailty score,^17^ comorbidities and medications on admission, risk factors for stroke, respiratory disease course, requirement for intensive care, laboratory/imaging results and modified Rankin score (mRS).^18^ The mRS was captured at two time points: at nadir and at discharge from hospital or the first follow-up assessment visit. The mRS was selected for several reasons. In view of the expected heterogeneity of neurological conditions, no single scale would have been considered optimal, and the consensus view was that the anticipated high proportion of strokes, familiarity of most clinicians with the mRS and the ease of its administration made the mRS the best candidate. The CRF was hosted on ALEA through the Clinical Information Research Unit at the University of Southampton. Data lock was 14 October 2020.

### Inclusion criteria

Physicians were invited to complete a CRF for any adult patient (≥18 years) hospitalized with a neurological or psychiatric presentation and COVID-19, or else developing these conditions whilst in hospital with COVID-19. Using World Health Organization criteria, cases were defined as ‘confirmed COVID-19’ if polymerase chain reaction (PCR) of respiratory samples or CSF was positive, or serology was positive for anti-SARS-CoV-2 antibodies. Cases were defined as ‘probable COVID-19’ if a chest radiograph or CT was consistent with COVID-19 but PCR and serology were negative or not done. Finally, cases were defined as ‘possible COVID-19’ if suspected on clinical grounds by the notifying clinician but PCR, serology and chest imaging were negative or not done,^2^ or if these data were unavailable. Cases of nosocomial infection following admission with a primary neurological presentation were excluded.

### Clinical case definitions

Patients were classified using standardized clinical case definitions.^2^^,^^19^ Cerebrovascular events were defined as symptoms, signs and/or neuroimaging consistent with transient ischaemic attack, ischaemic or haemorrhagic stroke, or intracranial venous thrombosis. Central inflammatory conditions were defined as those involving the CNS, with evidence of meningeal, parenchymal or vascular inflammation (CSF white cell count > 4/mm³, and/or protein > 0.45 g/dl, and/or neuroimaging consistent inflammation and/or demyelination).^2^ For psychiatric disorders, CRFs were assessed by a sub-specialty team of senior psychiatrists (co-authors TN and TP). Delirium was defined in accordance with the DSM-5 and the Ten Societies position statement^20^: (i) new-onset disturbance in attention, awareness and cognition, developing over hours or days, with some fluctuation, not in the context of a severely reduced level of arousal, such as coma, and not secondary to medication or substance misuse; and (ii) encephalopathy attributable to fever/sepsis, and/or hypoxia–ischaemia. Therefore, severe encephalopathy was defined as those with a severely reduced level of arousal (a Glasgow coma score ≤13/15 and/or seizures). Psychiatric presentations were considered a primary diagnosis if there was no evidence of an explanatory neurological disorder (e.g. psychosis without encephalitis/delirium). When multiple psychiatric diagnoses were reported, the primary diagnosis was ascertained in accordance with Bedford’s hierarchical model,^21^ which places psychiatric conditions in the following order of primacy: organic disorders (including neurocognitive disorder), followed by psychotic disorders, followed by mood disorders, followed by anxiety disorders, and finally personality/behavioural disorders. Peripheral neuropathies were cases involving the peripheral nervous system and categorized as inflammatory and non-inflammatory, on the basis of the reported diagnosis and whether inflammation is the sole recognized pathophysiological cause of this diagnosis; for example, Guillain–Barré syndrome is an archetypal inflammatory neuropathy, when compared to critical illness neuromyopathy.

When cases met multiple clinical case definitions, the primary definition was determined by blinded adjudication of the CRF data by three groups of senior authors representing neurology, psychiatry and stroke. Discrete clinical case definitions reported in the same patient were considered ‘overlapping syndromes’, for example, Guillain–Barré syndrome and an ischaemic cerebrovascular event. When complications were consistent with the primary clinical case definition, such as haemorrhage in acute haemorrhagic leukoencephalopathy, the primary diagnosis sufficed.

Patients with stroke were compared with those from the national stroke audit [Sentinel Stroke National Audit Programme (SSNAP)] over a comparable period in the preceding year (April—June 2019). Patients presenting with cerebrovascular events below the age of 60 were compared with those presenting above the age of 60.

### Statistical analysis

Statistical analysis was performed using SAS software (version 9.4; SAS Institute, Cary, NC, USA), SPSS v26 (IBM) and GraphPad Prism v8.4.3 (GraphPad Software, LLC). Normality of distribution was assessed using Kolmogorov–Smirnov tests. Data were analysed using descriptive statistics, group comparison tests, chi-squared tests, *z*-tests for independent proportions, and univariable logistic regression. A good outcome was defined as mRS ≤2 (reflecting no symptoms, slight disability, but independent) and a poor outcome as mRS >2 (moderate disability requiring assistance, or worse, including death). Multivariable logistic regression models were developed using baseline pre-COVID-19 variables with >80% data availability. Two sensitivity analyses were carried out for each model, one adjusting for diagnostic categories, and one using multiple imputation to account for the potential effect of missing data. The imputation model used a fully conditional specification and included the auxiliary variables weight and mRS at nadir. All hypothesis testing was two-tailed with alpha <0.05.

### Data availability

Study data are available from the authors subject to institutional agreements and ethical approvals.

## Results

### Demographic and clinical characteristics

Of 314 electronic CRF invitations accepted, 277 (89%) were submitted. The British Peripheral Nerve Society platform independently contributed an additional 24 cases. Cases not meeting the inclusion criteria or with incomplete core data were excluded (Supplementary Fig. 1). Included cases were from a broad range of sub-specialities and geographical distribution (Supplementary Figs 2 and 3).

Of 267 included cases, 95 (36%) were female, and 44 (18%) were from Black, Asian and minority ethnic groups (Table 1). 113 (42%) were below the age of 60 years. COVID-19 was confirmed or probable in 239 (90%) patients, with 28 (10%) defined as possible COVID-19 disease. The median (IQR) Rockwood frailty score before COVID-19 was 3 (2–5) (medical problems well controlled, but not regularly active beyond routine walking). Median (IQR) of Glasgow coma score on admission was 15 (14–15). Comorbidities were common, with 196 (81%) cases having at least one (Table 1). In addition, 66 (28%) had comorbid neurological disease, and 22 (10%) had a history of psychiatric illness. The most common non-neurological symptoms were fever (172, 73%), cough (139, 67%) and lethargy (124, 68%). Anosmia and/or ageusia was reported in 21 (18%) cases (Supplementary Table 2).

**Table 1:** Patient demographics and clinical characteristics

		All patients
Demographics		

Age in years, *n* (%)	20–29	6 (2)
	30–39	15 (6)
	40–49	35 (13)
	50–59	57 (21)
	60–69	51 (19)
	70–79	50 (19)
	80–89	36 (14)
	>90	17 (6)
Sex, *n* (%)	Male	172 (64)
	Female	95 (36)
Ethnicity, *n* (%)	Asian	23 (9)
	Black	21 (8)
	White	196 (73)
	Mixed	3 (1)
	Unknown	24 (9)
COVID diagnosis, *n* (%)	Confirmed or probable	239 (90)
	Possible	28 (10)

Clinical characteristics		

ICU admission, *n* (%)	Yes	76 (28)
	No	171 (64)
	Unknown	20 (8)
Ventilation required, *n* (%)	None	165 (62)
	NIV	15 (6)
	Invasive	67 (25)
	Unknown	20 (7)
Pre-COVID-19 frailty score, median (IQR)		3 (2–5)
At least one co-morbidity, *n* (%)		196 (81)
Type of co-morbidity, *n* (%)	Any neurological	66 (28)
	Any psychiatric	22 (10)
	Hypertension	125 (48)
	Diabetes mellitus	63 (24)
	Atrial fibrillation	43 (18)
	Congestive heart failure	19 (10)
	Previous TIA/stroke	25 (13)
Number of co-morbidities, median (IQR)		2 (1–4)
Admission GCS, median (IQR)		15 (14–15)
Fever, *n* (%)		172 (73)
Admission WCC, median (IQR)		8 (6–12)
Admission CRP, median (IQR)		41 (9–140)
Any non-neurological, non-respiratory systemic complication, *n* (%)		101 (42)
mRS at nadir, median (IQR)		4 (3–5)
mRS at outcome, median (IQR)		3 (2–5)
Improvement in mRS score, *n* (%)		125 (53)
Admission length in days, median (IQR)		23 (7–48)
Death, *n* (%)		57 (24)

mRS refers to modified Rankin Scale. Pre-COVID-19 frailty score refers to Rockwood frailty score. For definition of medically significant co-morbidities, see Supplementary methods. Improvement in mRS score was defined as mRS at outcome < mRS at nadir, or mRS score of 0 at both nadir and outcome.

### Overview of neurological and psychiatric conditions

**Figure 1:**
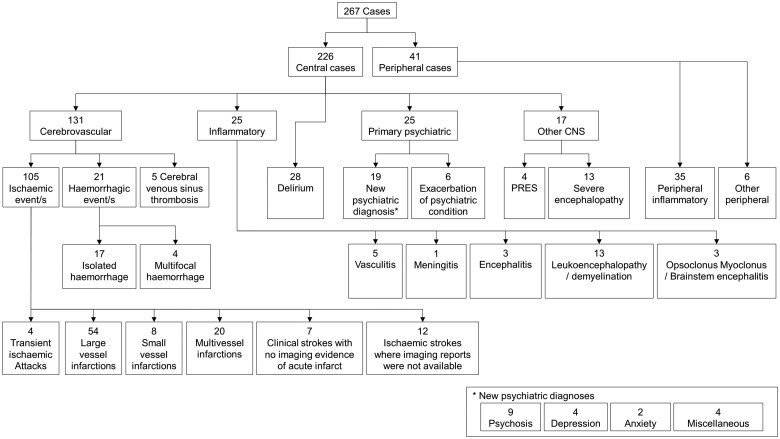
**Classification of main neurological diagnoses**.

**Figure 2:**
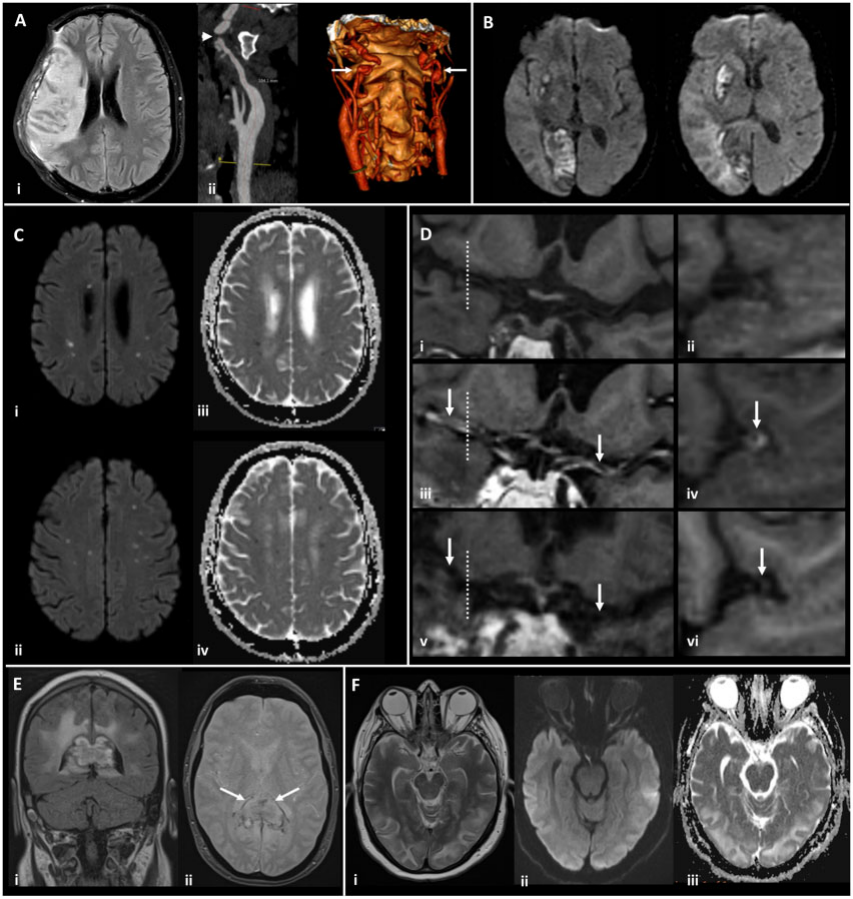
**Magnetic resonance imaging demonstrating the range of neurological complications seen in this study**. (**A**) Territorial infarct, secondary to internal carotid artery (ICA) dissection in a middle-aged previously fit male: Axial fluid-attenuated inversion recovery image (i) showing a right middle cerebral artery (MCA) territory infarct following decompressive craniectomy for malignant MCA syndrome despite treatment with thrombolysis. Reformatted images from a CT angiogram (ii) showing irregularity of the extracranial segment of both internal carotid arteries, consistent with dissection (arrows), with tight stenosis of the true lumen on the right (arrowhead). (**B**) Multiple territorial infarcts in a female >60 years old with hypertension and dyslipidaemia: Diffusion-weighted images (DWI) demonstrate recent infarcts in the right medial occipital lobe and lentiform nucleus, involving the territories of the right posterior cerebral artery and lenticulo-striate perforators of the right MCA respectively. (**C**) Acute lacunar infarcts due to small vessel vasculopathy in a male > 60 years old, with a background of hypertension and type 2 diabetes: B1000 images (i, ii) and corresponding apparent diffusion coefficient (ADC) maps (iii, iv) from DWI showing multiple tiny foci of restricted diffusion. (**D**) Vasculitis in a male >60 years old, with a background of type 2 diabetes, hypertension and hypercholesterolaemia: T_1_-weighted SPACE vessel wall imaging of both distal ICAs and proximal MCAs, with curved multiplanar coronal reconstructions along the course of both proximal MCAs (first column) and perpendicular to the right MCA (second column, at the position of the dotted line). Pre-treatment pre-contrast (i, ii) and post-contrast images (iii, iv) demonstrate abnormal concentric, long segment vessel wall enhancement (arrows) of both proximal MCAs. Post-contrast images after treatment with prednisolone and tocilizumab (v, vi) demonstrate treatment response with resolution of the previous abnormal mural MCA enhancement (arrows). (**E**) Acute encephalomyelitis with haemorrhage in a middle-aged male, with a history of chronic obstructive pulmonary disease, who required intensive care and haemofiltration: Coronal FLAIR (i) and axial gradient echo (ii) images showing focal heterogeneous signal abnormality and swelling of the splenium of the corpus callosum, with peripheral low signal indicative of haemosiderin staining (arrows). Confluent high signal is present in periventricular and deep white matter of the parieto-occipital region. (**F**) Typical imaging appearances of posterior reversible encephalopathy syndrome in a normotensive middle-aged female: Axial T2 image (i) demonstrating hyperintense signal in subcortical white matter of both occipital lobes, with B1000 image (ii) and ADC map (iii) from DWI showing no corresponding restricted diffusion.

Most cases primarily involved the CNS (226, 85%) (Fig. 1). The largest group were cerebrovascular events, comprising 131 (49%) patients (Figs 1 and 2). The second most common CNS groups were delirium (28, 11%) and central inflammatory conditions (25, 9%); the latter comprising mostly demyelination and leukoencephalopathy, but also vasculitis, encephalitis, and opsoclonus–myoclonus syndrome (Figs 1 and 2). Psychiatric presentations (25, 9%) were most commonly new diagnoses (19, 76%) but included six patients with an exacerbation of an underlying condition (24%). Those remaining were all other encephalopathies (17, 7%), including 13 with severe encephalopathy and four with posterior reversible encephalopathy syndrome. The peripheral nervous system was primarily involved in 41 (15%) cases, of which 35 (85%) were inflammatory and six (15%), were non-inflammatory.

### Multiple overlapping diagnoses

**Figure 3:**
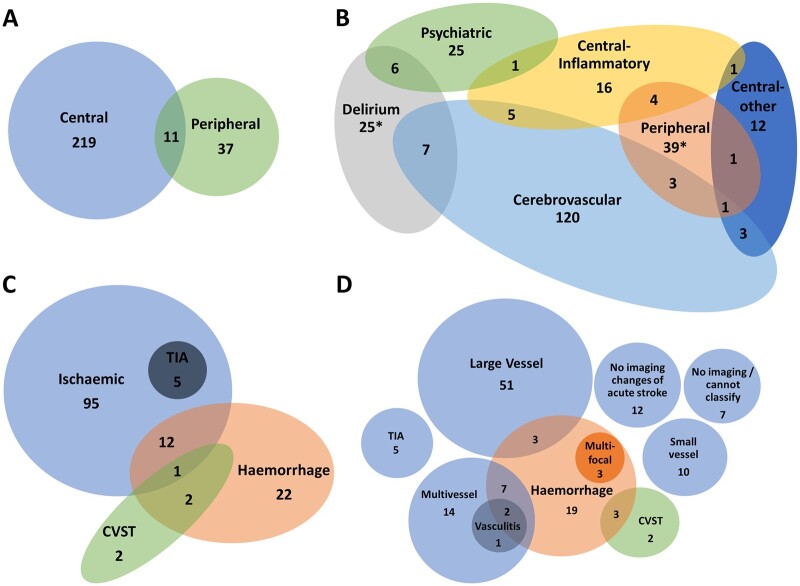
**Venn diagrams showing overlap of diagnostic groups.** The numbers shown here are when all diagnoses were considered, in addition to the primary neurological diagnosis. The total numbers for several groups are larger in this Figure than the primary diagnosis flowchart (Fig. 1) due to coexisting diagnoses. (**A**) Central and peripheral nervous system disease. (**B**) Primary diagnostic categories (*two cases of Guillain–Barré syndrome with delirium were not possible to accommodate on this diagram). (**C**) Stroke group subtypes. (**D**) Specific stroke group subtypes. CVST, cerebral venous sinus thrombosis; TIA, transient ischaemic attack.

A proportion of patients (34, 13%) met multiple primary clinical case definitions, with each diagnostic group overlapping at least two others, and 11 cases (32%) involving both the CNS and peripheral nervous system (Fig. 3A and B). The greatest overlap was in the cerebrovascular (19 cases, 14%), delirium (15, 40%) and central inflammatory (11, 4%) groups. Patients with overlapping presentations more frequently required intensive care (20, 65% versus 56, 26%, *P* < 0.001) and ventilation (71% versus 28%, *P* < 0.001) compared to those meeting a single clinical case definition.

### Cerebrovascular disorders

**Figure 4:**
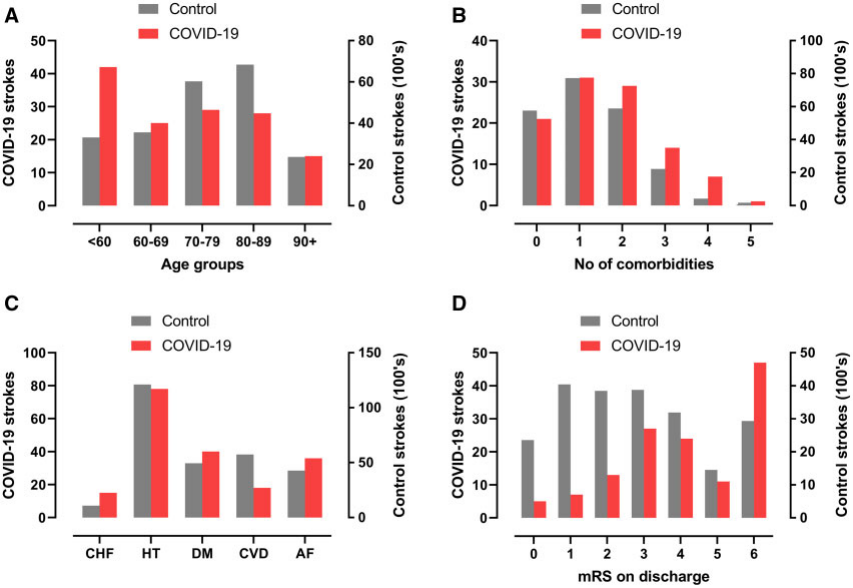
**COVID-19 strokes versus historical controls.** Comparison between strokes associated with COVID-19 in this study and strokes from a national UK audit in 2019. (**A, B**) total number of co-morbidities which are risk factors for stroke (atrial fibrillation, hypertension, diabetes mellitus, congestive heart failure and previous TIA or stroke). (**C**) Age distributions. (**D**) mRS (modified Rankin scale) scores on discharge from hospital (or death).

Most primary cerebrovascular events were ischaemic (105, 80%), including large vessel occlusions, small vessel infarcts and multi-territory infarcts affecting both large and small vessel distributions. Most cases of intracerebral haemorrhage were isolated (17, 81%), but four (19%) were multifocal, and there was considerable overlap with other clinical case definitions, especially multi-vessel strokes (Fig. 3C and D). Patients with cerebrovascular events had a higher frequency of non-CNS thrombotic complications (e.g. pulmonary embolism, cardiac thrombus, renal artery thrombosis) than the rest of the cohort (11% versus 5%). As compared to historical non-COVID-19 stroke patients, those in association with COVID-19 were younger, had a greater number of comorbidities, and cerebrovascular risk factors (especially, diabetes mellitus, congestive heart failure, and atrial fibrillation), and had a worse outcome (Fig. 4).

Within our cohort, cerebrovascular events occurred in 35 (27%) patients aged <60 years and, relative to those aged >60 years old (96, 73%), they presented later, with a median (IQR) onset after respiratory symptoms of 10 days (0–18) compared to 0 days (−7 to 7) (*P* < 0.001). The younger group also had lower rates of co-morbidities increasing stroke risk (16, 67% versus 77, 88%), a higher proportion of multi-vessel occlusion (9, 31% versus 11, 15%) and more non-neurological thrombotic events (6, 18% versus 8, 8%) (Supplementary Table 3).

### Central nervous system inflammatory conditions

The most common complication in the central inflammatory group was leukoencephalopathy, affecting 13 (52%) cases. Encephalitis was reported in three; in one PCR of CSF was positive for SARS-CoV-2. Nine cases (43%) needed ventilation and had acute kidney injury, of which seven (78%) required renal replacement therapy.

### Delirium

Delirium had a bimodal age distribution, the first peak at 30–39 years (4, 14%) (Supplementary Table 4). Relative to the rest of the cohort delirium was not significantly associated with established risk factors, such as age, markers of systemic inflammation and intensive care (Supplementary Table 5). There were six cases that met both delirium and psychiatric diagnostic criteria, of which three were <60 years old. One presented with new onset paranoid beliefs 48 h prior to delirium; one had profound anxiety progressing to Capgras syndrome (a delusion of misidentification); and one developed prominent hallucination requiring multiple antipsychotic medications with ongoing symptoms several months after systemic recovery.

### Severe encephalopathy

There were 13 additional cases of severe encephalopathy, that did not meet a clinical case definition of delirium as they had a severely reduced level of arousal.^20^ These severe encephalopathies were characterized by significant complications, frequently affecting consciousness, namely: provoked seizures and status epilepticus in younger patients with no premorbid conditions, cardiac and renal complications including cardiac arrest in working-age adults, and seizures in older adults with significant pre-existing neurological comorbidities (Supplementary Table 6). Those with this severe encephalopathy (*n* = 13), in comparison to delirium (*n* = 28), were younger (median decade 50–59 versus 60–69 years), had higher rates of admission to intensive care (8, 62% versus 8, 29%) and ventilation (8, 67% versus 9, 33%) and a longer median (IQR) duration of ventilation of 11 (0–36) versus 0 (0–13) days.

### Psychiatric diagnoses

New psychiatric diagnoses included nine cases of psychosis, four cases of depression, two cases of anxiety and a single case each of catatonia, mania, neurocognitive/dementia-like syndrome and functional neurological disorder.

### Peripheral neuropathies

The peripheral neuropathies reported were predominantly Guillain–Barré syndrome. Non-inflammatory peripheral neuropathy cases were mostly critical illness neuromyopathies, albeit without neurophysiological confirmation. There were no deaths in any patients with peripheral neuropathy.

### Timing of neurological symptoms

**Figure 5:**
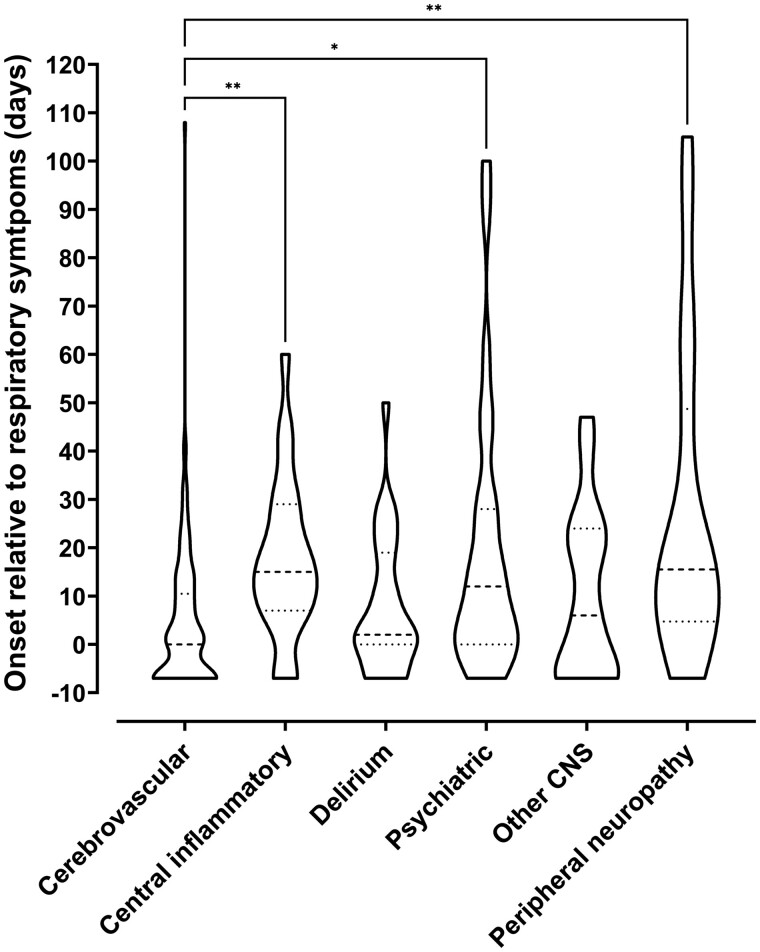
**Timing of onset of neurology.** Violin plot demonstrating distributions of time intervals in days between onset of respiratory symptoms and onset of neurological symptoms for each primary diagnostic category. Patients whose neurological symptoms preceded COVID-19 symptoms were arbitrarily assigned a value of minus seven days. The Kruskal–Wallis test was used to determine any significant difference in time intervals between groups (*P* < 0.0001). Dunn’s multiple group comparison test showed a significant difference between stroke and central inflammatory primary diagnostic groups (*P* = 0.001), stroke and psychiatric groups (*P* = 0.037), and stroke and peripheral groups (*P* = 0.003).

In 66 (47%) patients, the onset of neurological disturbance occurred after their respiratory condition improved, and in 69 (29%), the neurological symptoms predated the onset of COVID-19 symptoms. Neurological symptoms started after a median (IQR) of 12 (2–22) days following onset of respiratory symptoms and lasted for a median (IQR) of 20 days (6–44) (Fig. 5). Cerebrovascular events were associated with the earliest onset, with median (IQR) time from respiratory symptom onset to cerebrovascular event of 7.5 (2–16) days. Interestingly, longer time to onset was observed in the central inflammatory, psychiatric and peripheral neuropathy diagnostic categories.

### Clinical outcome and risk factors

Outcome mRS was assessed at a median (IQR) follow-up time of 30 days (7–60). This was at hospital discharge (48%), as an inpatient (22%) or an outpatient (29%). Patients in this study were substantially disabled, since 131 (56%) had an outcome mRS of 2–5; moreover, 57 (24%) patients died. Outcome was assessed in three ways: whether mRS improved (mRS at outcome versus mRS nadir), mRS at outcome and death.

**Figure 6:**
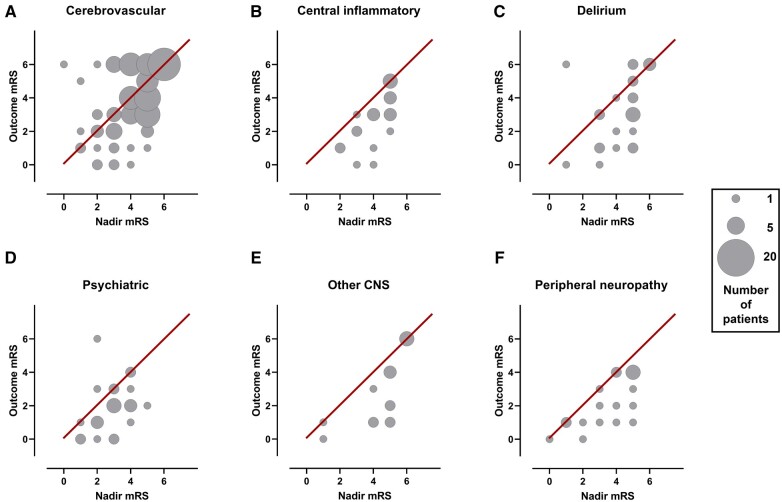
**Recovery from neurological condition.** Bubble plots displaying the relationship between mRS (modified Rankin scale) at nadir of illness whilst in hospital and mRS at outcome assessment, within individual diagnostic categories. Bubble area corresponds to patient number. Line of equivalence is shown in red: cases below the line improved, cases above the line got worse, while cases on the line stayed the same.

Improvement in outcome mRS relative to the mRS score at nadir of illness was seen in all primary diagnostic categories other than cerebrovascular events (Fig. 6). There was a significant difference in mRS improvement across diagnostic groups (Supplementary Table 7, *P* < 0.001). Cerebrovascular events improved the least (39%, *P* < 0.001), while central inflammatory conditions improved most (77%, *P* < 0.03).

Multivariable analysis using baseline variables easily available at admission, demonstrated a higher probability of a poor outcome (mRS ≤2) with older age, a higher Rockwood frailty score and higher white cell count on admission. In comparison, the association of outcome with individual neurological diagnostic categories was negligible. A similar pattern was observed with mortality (Table 2).

**Table 2:** Clinical outcome

	Complete case model: variables available at admission		Complete case model: adjusting for diagnostic variables		Multiple imputation model	
	OR (95% CI)	*P*-value	OR (95% CI)	*P*-value	OR (95% CI)	*P*-value
Outcome variable: MRS score at outcome >2						

Age (10-year age groups)	1.67 (1.26, 2.22)	<0.001	1.66 (1.23, 2.25)	0.001	1.64 (1.28, 2.11)	<0.001
Sex at birth (Male)	1.61 (0.73, 3.55)	0.236	1.40 (0.61, 3.24)	0.431	1.74 (0.86, 3.52)	0.124
Non-white ethnic group	1.54 (0.62, 3.86)	0.355	1.73 (0.66, 4.55)	0.267	1.50 (0.64, 3.48)	0.347
Clinical frailty scale (Rockwood)	1.51 (1.13, 2.02)	0.005	1.48 (1.08, 2.03)	0.014	1.49 (1.16, 1.92)	0.002
Pre-existing neurological disease	1.05 (0.39, 2.87)	0.920	1.38 (0.47, 4.10)	0.560	1.45 (0.58, 3.58)	0.425
Hypertension	0.70 (0.30, 1.65)	0.418	0.75 (0.31, 1.81)	0.517	0.68 (0.32, 1.48)	0.333
Diabetes	1.16 (0.46, 2.98)	0.751	0.96 (0.36, 2.55)	0.928	1.73 (0.74, 4.00)	0.203
Log_10_ white cell count at admission	7.51 (1.20, 46.92)	0.031	6.56 (1.01, 42.53)	0.049	6.62 (1.31, 33.58)	0.023
Cerebrovascular event diagnosis			2.84 (0.72, 11.22)	0.136		
Central inflammatory diagnosis			1.68 (0.39, 7.33)	0.490		
Delirium diagnosis			0.94 (0.24, 3.67)	0.932		
Psychiatric diagnosis			0.65 (0.13, 3.26)	0.600		
Other encephalopathy diagnosis			0.94 (0.16, 5.70)	0.950		
Peripheral neuropathy diagnosis			2.45 (0.47,12.87)	0.289		

Outcome variable: patient death						

Age (10-year age groups)	1.50 (1.12, 2.00)	0.007	1.42 (1.06, 1.91)	0.020	1.48 (1.14, 1.92)	0.003
Sex at birth (Male)	1.13 (0.50, 2.58)	0.762	1.17 (0.50, 2.75)	0.714	1.31 (0.63, 2.70)	0.473
Non-white ethnic group	1.84 (0.53, 6.34)	0.334	1.82 (0.52, 6.40)	0.353	1.39 (0.45, 4.25)	0.566
Clinical frailty scale (Rockwood)	1.65 (1.28, 2.13)	<0.001	1.56 (1.20, 2.04)	0.001	1.54 (1.23, 1.93)	<0.001
Pre-existing neurological disease	0.62 (0.24, 1.59)	0.318	0.79 (0.30, 2.08)	0.630	0.89 (0.39, 2.06)	0.789
Hypertension	0.56 (0.24, 1.30)	0.179	0.58 (0.25, 1.38)	0.219	0.47 (0.21, 1.03)	0.059
Diabetes	1.06 (0.43, 2.62)	0.905	0.98 (0.39, 2.48)	0.964	1.77 (0.78, 3.97)	0.170
Log_10_ white cell count at admission	1.76 (0.33, 9.35)	0.507	1.46 (0.24, 8.74)	0.677	2.35 (0.55, 10.02)	0.249
Cerebrovascular event diagnosis			2.09 (0.58, 7.52)	0.262		
Delirium diagnosis			0.84 (0.22, 3.26)	0.798		
Psychiatric diagnosis			0.48 (0.05, 5.05)	0.544		
Other encephalopathy diagnosis			0.74 (0.07, 8.08)	0.803		

Multivariable logistic regression analysis of mRS score at outcome >2 and patient death. Patients could have multiple diagnoses. Inflammatory and peripheral neuropathy diagnoses were excluded from the patient death analysis, as no deaths occurred in these groups.

## Discussion

Through a nationwide surveillance study of adults hospitalized with COVID-19, conducted through a cross-specialty collaboration spanning six national physician associations, we present the broad spectrum of potential neurological and psychiatric complications of COVID-19, across central and peripheral nervous systems. Our results build on existing knowledge,^1^^,^^4–8^^,^^10–12^^,^^22^ by applying standardized, internationally agreed, *a priori* clinical case definitions and independent, blinded case adjudication to determe specific diagnostic group membership, and by presenting detail on the overlap between clinical presentations. We provide further evidence of a coagulopathy precipitating stroke in young patients, occurring in the para-infectious phase of illness, and suggest this group is distinct to older patients with multiple conventional risk factors. Nevertheless, despite a younger cohort of patients with COVID-19 associated stroke compared to non-COVID-19 stroke patients, conventional, often modifiable, risk factors were more frequent even in younger patients.

### Timing of onset

Onset of neurological disease, in days relative to respiratory symptoms, varied across different diagnostic categories. In 29% of cases, neurological symptoms preceded respiratory symptoms, suggesting occurrence during the virological, or para-infectious phase, the early part of which is usually asymptomatic.^23^ This supports early mechanisms, such as activation of the innate immune system and direct viral effects on endothelial cells. Within the context of a pandemic, neurological syndromes described in this study could be a sentinel sign of COVID-19, and we encourage SARS-CoV-2 testing of patients with neurological presentations, including acute encephalopathy, in settings where asymptomatic testing is not routine. The later presentation of central inflammatory and peripheral nerve presentations, after respiratory recovery, and the high rates of improvement seen in these groups, supports a post-infectious process, driven by an adaptive immune response.

### Possible mechanisms underlying peri-COVID-19 stroke

Our comparison with pre-COVID-19 SSNAP data identified higher rates of young stroke in our COVID-19 cohort, despite reports of a reduction in overall stroke admissions during the pandemic.^24^ The underlying mechanisms leading to stroke may differ between younger and older cases, as younger strokes had a significantly delayed presentation, were associated with fewer comorbidities, and demonstrated higher rates of both multi-vessel occlusion and of thrombotic complications outside of the CNS. These findings are supportive of a para-infectious thrombo-inflammation, potentially driven by endothelitis and subsequent cytokine release, and in line with previous reports of elevated serum markers of coagulopathy in stroke patients.^25^ Early administration of anti-inflammatory therapy has potential benefit, and our data strengthen the case for trials to consider stroke outcomes so that this effect can be evaluated. In older cases, where the highest risk is in the initial days of symptoms, it is likely that COVID-19 is precipitating stroke similarly to other acute respiratory infections, through interaction with existing cerebrovascular risk factors.^26^

### Encephalopathy

Encephalopathy is widely reported in COVID-19^7^^,^^27^^,^^28^ and, in an undifferentiated form, has been demonstrated to be an independent predictor of death and poorer functional recovery in survivors.^9^^,^^29^ However, there is a lack of consensus as to the distinct underlying pathophysiology.^30^ The presentation of delirium in younger patients, seen frequently in our cohort, is unusual for a respiratory illness in the absence of severe hypoxia, and suggests COVID-19 confers additional risk compared to other infections. In addition to delirium, we identified distinct aetiological groups, including posterior reversible encephalopathy syndrome and a severe encephalopathy outside the accepted definition of delirium.^20^ This latter syndrome may represent excitotoxic injury, such as is seen following seizures, metabolic disturbance, or an underlying inflammatory or microvascular process. The greater need for intensive care in this group may represent both the cause (exposure to potentially ictogenic medications) and the consequence of seizures. Indeed, multiple overlapping disease mechanisms may be apparent, even within an individual patient, and further studies are underway to evaluate this (COVID-CNS).

### Clinical outcome

Age and a higher Rockwood frailty score were much more indicative of outcome than the neurological or psychiatric disorder. It is interesting that the adjusted hazard ratio of age for outcome (death or hospitalisation) is 10-fold that of neurological disorders.^31^ Individual disease groups were heterogeneous and did not demonstrate significantly different outcomes, but this requires further study in larger cohorts. Ongoing assessment of the predictive power of premorbid frailty will be important as we see increasing numbers of young people affected. Poor outcome also associated with a high admission white cell count, which might be a useful predictor given that this is usually normal in the early stages of COVID-19.^32^

### Limitations

This study has several limitations. It concerns a specific population of COVID-19 positive patients requiring admission to hospital because of neurological or psychiatric conditions or else developing these complications whilst in hospital with COVID-19. Hence it would not have captured neurological symptoms, such as headache, anosmia, dysgeusia and mild cognitive dysfunction, generally looked after in the community or in outpatient services. Although this study captured data from a breadth of different specialties, there was still under-representation of psychiatrists, primary care and internal medicine which may have skewed the study population to more severe cases. The mRS was used as a clinical outcome measure but this scale has not been validated for its measurement properties across the wide spectrum of conditions studied here. We did not review primary clinical data held locally by the referring physician, and could not perform an independent review of data submission quality. Where possible the original syndromic classification from the referring team was presumed to be correct. On rare occasions, there was a reclassification of cases based on senior author panel review.

Participation by physicians occurred during an unprecedented healthcare and social emergency, during which clinical service and research teams were stretched. This has the potential for reporting bias, in particular under-reporting of mild disease, and potential over-representation of unusual presentations. These circumstances are also likely to have contributed to missing clinical data fields (Supplementary Table 8). Although not the largest study to date,^6^ this study provides both granularity of detail and breadth of sub-specialty input. We included cases from the very beginning of the pandemic and PCR confirmation of COVID-19 was not always present, though COVID-19-associated neurology was an inclusion criterion.

The SSNAP database is a high-quality rolling national audit, which captures routine, consecutive, unselected stroke cases and has a low risk of bias; there is good overlap with essential data fields in our study since SSNAP is so comprehensive. We selected a SSNAP database time period from the year preceding the pandemic, which was comparable both in season and duration to our study. However, the comparability of these two cohorts has to be guarded with caution, due to the potential for selection biases in our cohort. The fact that our findings were similar to those of the SETICOS study,^33^ which used a case–control design, provides some validation of the approach used here.

## Conclusions

In summary, this nationwide, cross-specialty study of neurological and psychiatric manifestations of COVID-19, has identified older age and a higher pre-COVID-19 frailty score to be associated with poor outcome, and the effect of these baseline characteristics overshadowed the effects of specific neurological diagnoses. Presentations spanned pre-symptomatic, early and later phases of COVID-19, implying different pathophysiological processes may occur, and these may act synergistically in driving neurological complications. Cerebrovascular events were the most common complication and, in young as opposed to older patients, COVID-19-associated events occurred later after respiratory symptom onset, supportive of thrombo-inflammation and systemic coagulopathy, and this requires further study. A severe encephalopathy beyond the clinical definition of delirium occurs during COVID-19. Future work must focus on longer term follow up of specific disease groups, and mechanistic studies using neuroimaging and biosamples to better characterize pathophysiology.

## Supplementary material

Supplementary material is available at *Brain Communications* online.

## Supplementary Material

fcab168_Supplementary_Data

## Appendix

For the purposes of this paper, the *Coro*Nerve Studies Group includes all the authors and Nisha Abraham-Thomas, Katja Adie, Claire Allen, Nabeel Amiruddin, Heather Angus-Leppa, Fahim Anwar, Neil Archibald, James Arkell, James Armitage, Cherie Armour, Peter Arthur-Farraj, Marc Atkin, Mark Baker, Nawar Bakerly, Rajaram Bathula, Alexandra Belcher, Benson Benjamin, Viraj Bharambe, Gordon Blair, Catrin Blank, Jim Bolton, Michael Bonello, Iryna Boubriak, Claire Boynton, David Breen, Robert Brenner, Dennis Briley, Fiona Brodie, Helga Brown, Stefania Bruno, Ed Bullmore, Angus Butchart, Hannah Castell, Kah Lok Chan, Bharath Cheripelli, Patrick Chinnery, James Choulerton, Philip Clatworthy, Joanne Clements, Jonathon Coates, Alasdair Coles, Ceryce Collie, Gwen Collin, Peter Cottrell, Charles Coughlan, Sarah Crisp, Mazen Daher, Jane Dale, Ruth Davies, Sylviane Defres, Sofia Dima, Katherine Dodd, Liam Dodge, Fergus Doubal, Cordelia Dunai, Ahilanadan Dushianthan, Dipankar Dutta, Richard Ellis, Salwa Elmamoun, Hannah Emerson, Bethany Facer, Patricia Fearon, Peter Fernandes, Barnaby Fiddes, James Firth, Emma Fisher, Leonora Fisniku, Alasdair Fitzgerald, Enrico Flossman, Caroline Fornolles, Andrew Gallagley, Brian Gallen, Garcia del Carrizo Fernando, Alan Gemski, Jackie Gilbert, Effrossyni Gkrania-Klotsas, Farhad Golestani, Bruno Gran, Andrew Gratrix, Susan Green, Helen Grote, Rebecca Grue, Sabine Grundler, Alexander Grundmann, Savini Gunatilake, Mahir Hamad, Khalid Hamandi, Hisham Hamdalla, Kirsty Harkness, Neil Harrison, Timothy Harrower, Jennifer Hartman, Ahamad Hassan, Ghaniah Zeb Hassan-Smith, Alison Hatfield, Catherine Hatfield, Charles Hillier, Akram Hosseini, Gary Hotton, Jack Hubbett, Saif Huda, Nathan Huneke, Caroline Hutchison, Anne-Catherine Huys, Thomas Ian, Hajira Iftikhar, Ihmoda Ihmoda, Muhammad Ilyas, Sissi Ispoglou, Tom Jenkins, Nicola Jones, Ingrid Kane, Ryan Keh, Hind Khalifeh, Jeff Kimber, Amit Kishore, Martin Knolle, Christopher Kobylecki, Sander Kooij, Anita Krishnan, Matthew Lambert, Gavin Langlands, Phil Laws, Charles Leek, Lucia Li, George Lilly, Rebecca Luxton, Graham Mackay, Barbara Madigan, Melissa Maguire, Azer Majeed, Arshad Majid, Gauhar Malik, Mark Manford, Richard Marigold, Sarah Marrinan, Monica Marta, Paul Matthews, Michael McCormick, Marcia McDougall, Caroline Mcinnes, David McKee, Isabel McMullen, Brian Menezes, Stephanie Miers, Thomas Minton, Amulya Misra, Dipak Mistry, James Mitchell, Hartmann Monika, Mireia Moragas, Hamish Morrison, Walied Mowafi, Paul Mudd, Louis Murphy, Anna Nagy, Edward Newman, Choo Ng, Sam Nightingale, Khin Nyo, Suzanne O’Neil, Richard O'Brien, Ivy Ong, Matt Oram, Belgin Ozalp, Stella-Marie Paddick, Kevin Pankhurst, Nehal Parmar, Carmen Parr, Kath Pasco, Sarah Pearce, Gordon Plant, David Price, Gary Price, Jane Pritchard, Nicholas Pritchard, Harald Proeschel, David Protheroe, Terence Quinn, Akansha Rajan, Jessica Redgrave, Rebecca Redwood, Chris Rickards, Christina Roffe, Jonathan Rogers, Alia Roof, Amrit Sachar, Neshika Samarasekera, Amal Samaraweera, Stephen Sawcer, Shona Scott, Lakshmanan Sekaran, Jordi Serra-Mestres, Kanchan Sharma, Pamela Shaw, Mohammed Siddiqui, Emily Simon Thomas, Fai Lam Chun Chiang Sin, Andrew Sissons, Mara Sittampalam, Anushta Sivananthan, Thandar Soe, Mohamed Soliman, Andreas Sotiriou, Aginor Spanoulis, Jon Stone, James Sun, Peter Swann, Hafiz Syed, Konrad Szewczyk-Krolikowski, Leonie Taams, Amr Tageldin, Maryam Talaei, Emma Tallantyre, Riffat Tanveer, Lisa Templeton, Philip Thomas, Catherine Trezise, David Turner, Jaap van der Boom, Elizabeth Varghese, Angela Vincent, Briony Waddell, Tom Walker, Stephen Webb, Nic Weir, Chris Wharton, Lou Wiblin, Malcolm Wiggam, Tim Williams, Greta Wood, Nicholas Wood, Charmaine Yam.

## Abbreviations

CRFs =: Case Record Forms
IQR =: interquartile range
mRS =: modified Rankin score
SSNAP =: Sentinel Stroke National Audit Programme
